# Influence of Extruder’s Nozzle Diameter on the Improvement of Functional Properties of 3D-Printed PLA Products

**DOI:** 10.3390/polym14020356

**Published:** 2022-01-17

**Authors:** Piotr Czyżewski, Dawid Marciniak, Bartosz Nowinka, Michał Borowiak, Marek Bieliński

**Affiliations:** Department of Manufacturing Techniques, Faculty of Mechanical Engineering, Bydgoszcz University of Science and Technology, Kaliskiego 7, PL 85796 Bydgoszcz, Poland; dawid.marciniak@utp.edu.pl (D.M.); bartosz.nowinka@pbs.edu.pl (B.N.); michal.borowiak@roma.torun.pl (M.B.); marek.bielinski@pbs.edu.pl (M.B.)

**Keywords:** FFF method, PLA, extruder nozzle diameter, mechanical properties, macrostructural analysis

## Abstract

The dynamic growth of the use of polymer construction parts manufactured individually and in a small series makes it necessary to improve additive methods in the areas of materials, equipment and processes. By observing selected phenomena occurring during the processing of polymer materials in other production technologies (e.g., extrusion and injection molding), it is possible to obtain solutions that positively affect the final performance properties of the products obtained in additive manufacturing technologies using thermoplastic filament. The aim of this research was to determine the effect of the diameter of the print head nozzle on the spatial structure (path width) and selected mechanical properties of samples produced by the FFF method with PLA material. The obtained results were compared to the samples with a solid structure produced using injection molding technology. In the experiment, the RepRap device for additive manufacturing was used, with the use of nozzles with diameters of 0.2 mm, 0.4 mm, 0.8 mm and 1.2 mm. The test objects were produced with a layer height of 0.2 mm, full filling (100%) and with constant remaining printing parameters. The conducted research allowed us to conclude that the use of layer heights lower than the standard ones gives favorable results for selected mechanical properties. The use of an extruder nozzle diameter of 0.8 mm allows one to obtain a macrostructure with a high degree of interconnection of layers and paths and favorable mechanical properties. The test results can be used in the construction of functional elements that are produced by fused deposition modeling (FDM) and fused filament fabrication (FFF) methods in prototype, unit and small-lot production.

## 1. Introduction

Among the methods of plastics processing, technologies such as injection molding (IM), extrusion, thermoforming and 3D printing can be distinguished. These technologies vary in many aspects, but the common features of all of them is a high temperature and the processed material. Plastic deposition (FDM/FFF) commonly known as 3D printing is one of the additive manufacturing techniques. The process is based on the extrusion of plasticized polymeric material and its selective dosing through a nozzle to produce the designed geometry of the final product [1]. Most parts manufactured with the use of FFF are used as prototypes, design visualizations and educational models, but they are increasingly being used as finished products with utility purposes [2]. The growing interest in unit and small-batch production determines the development of this technology, including the design of the products for a specific manufacturing technique, e.g., FDM and FFF. This allows for the better use of the advantages, as well as reducing the limitations, of this technology [3]. Therefore, it becomes necessary to expand the possibility of predicting the behavior of the structural elements in operating conditions [4].

One of the most widely used materials in this process is polylactide (PLA). The main advantage of PLA is the facility of its processing. This also applies to professional printing machines as well as to cheaper additive manufacturing devices. The processing of PLA does not require heating of the working platform or a closed printing chamber, and it exhibits little processing shrinkage. For these reasons, polylactide is one of the most frequently modified plastics used in 3D printing. The following additives for PLA are used: wood flour, ceramics, metal, carbon fiber, glass fiber and many others [5].

The layered nature of the elements produced by FDM/FFF technology results in significant differences in the structure of the manufactured elements in relation to other technologies. A clear anisotropy of the mechanical properties in individual sections of the tested samples can be observed, which leads to the deterioration of strength in the direction perpendicular to the constructed layers [6]. Interference with the structure of layers and its modification are possible with the use of open-source software to divide the model into layers as demonstrated in the work of the Kreiger team. This procedure allows one to obtain more favorable performance indicators of the elements manufactured by FFF, mainly due to the greater flexibility in the selection of the technological parameters [7]. An example is Simplify3D software, which allows for the online adjustment of process parameters, i.e., temperature (chamber, platform, material plastification and layer deposition area) and printing speed (head movement and material extrusion) [8,9].

The parameters of the 3D printing process have the most significant influence on the properties of the components produced by FDM/FFF technology [10,11,12]. For this reason, the most important relationships between the various printing parameters and their influence on the strength of the final components should be determined.

Increasing the requirements concern, on the one hand, the increase in process efficiency and, on the other hand, the improvement of 3D printing quality. Łyżwa [13] describes the dependence of the process efficiency on the filling density, height of the printed layers and printing rate. Additionally, the quality of the prints was analyzed. The changes in the filling density influence the degree of filling of the model structure, which varies from 0%—empty object (shell)—to 100%—full object. An increase in the degree of filling causes a several-fold increase in both the processing time and material consumption. Increasing the efficiency of the 3D printing process is also associated with the need to increase the wear resistance of the head, which is solved, among others, by using a ruby tip in the nozzle (Olsson Ruby). The aluminum oxide used in this case is characterized by a high hardness, which additionally allows the processing of materials containing fibers or metal fillers [14]. 

One way to improve process efficiency is to increase the height of a single print layer. The layer thickness range for most FFF printers is typically between 0.06 mm and 0.4 mm. The studies show that a three-fold increase in layer height results in about a four-fold reduction in printing time [15].

In order to obtain more precise parts, low values of print layer thicknesses are applied, while structures printed with the use of a large layer thicknesses are characterized by a visible, striped sidewall structure. Moreover, round or inclined parts are deformed. A factor that has a large influence on a wide range of performance characteristics of the final product is the contribution of air voids inside the structure of a part. The following methods are mainly used to determine this factor: scanning electron microscopy, computed micro-tomography and optical microscopy. Bączkowski et al. determined the relationship between the content of air voids at the boundaries of the glue joint paths and its impact strength. For this purpose, images obtained with a Keyence microscope were analyzed [16].

Important results for PLA were also obtained by Kuznetsov and his team. The research included, among others, variable nozzle diameters, layer thickness and printing rates, which influenced the obtained structure. The strength parameters in relation to the obtained structure (contribution of air voids) were analyzed. As a result, a beneficial effect of using large nozzle diameters (0.8 mm) while maintaining a small layer thickness (0.15 mm) on the strength coefficients was proved [17].

The work of Sukindar et al. focused on the effect of the nozzle diameter on the pressure drop, geometric error and extrusion time. The conducted analysis showed that the diameter of the nozzle has a significant influence on the pressure drop in addition to the plasticizing chamber, which influences the consistency of the applied width of the path and, thus, the quality of the product finish. The highest pressure drop, which is not within the optimal range, was obtained with the use of a 0.2 mm nozzle [18].

In order to ensure the favorable performance of printed products, the layer height should be adjusted to the diameter of the nozzle. This procedure affects the value of interlayer cohesion [19]. 

A widely accepted mathematical model is used to determine the optimum printed layer height:

h≤0.75×D

where the recommended layer height is 0.5 *D* [20,21].

Nevertheless, the continuous development of 3D printing technology and the newer applications of printed parts make it necessary to search for new properties resulting not only from the material but also from the applied technology, e.g., by selecting non-standard process parameters.

Understanding the influence of the stability of the cooling parameters on the correct course of the process of applying successive layers is also an important area requiring thorough analysis. One of the important aspects is the uniform distribution of internal stresses related to the uneven course of the process [22].

Studies show that there is a value of the critical temperature range for a given degree of crystallinity of polylactide [23]. In the work of Wittbrodt [24], the relationship between the degree of crystallinity of polylactide and the processing temperature was featured. The processing of PLA with the use of a temperature equal to 210 °C provided a crystalline phase contribution of 14%, while in the case of 190 °C or 215 °C, this value was less than 6%. In the case of injection-molded parts, annealing the samples at 105 °C for 90 min improved the mechanical properties, including the flexural and tensile strength, Izod impact strength and heat resistance. These results confirm the significant effect of heat treatment on the processing properties of PLA [25,26,27].

In the case of determining the properties of polymeric materials, injection-molded specimens are the most frequently used. In the case of samples prepared by filament processing, the true nature of the polymer–gas structure is not fully reflected by the properties determined.

The aim of the present research was to assess the effect of a non-standard layer height equal to 0.2 mm (lower than that recommended by the state of the art) and selected diameters of the working head nozzle on the non-solid printed structure (geometric features of the layers, paths and air voids), as well as selected mechanical properties. The mechanical properties were related to the solid material obtained using the injection technology. The experiment was conducted with the use of standardized samples, appropriate for thermoplastic materials. In the conducted experiment, the functional properties of the analyzed samples were sought while maintaining constant process parameters (linear speed of the head and layer height). Due to the non-solid (porous) structure of the printed test objects, the results of the selected mechanical parameters were related to the material density. Changing the diameter of the nozzle allows one to obtain a repeatable degree of plasticization of the applied material. In order to provide full plasticization of the filament, despite the changing cross-section of the applied path (change in the path width), the proper parameters were selected, while changes in other process parameters were avoided. The low layer thickness gives the possibility of applying paths in the form of stripes, which results in fewer connections between adjacent paths inside a single layer and a larger contact area between the paths of subsequent layers.

## 2. Materials and Methods

### 2.1. Research Objects

**Figure 1:**
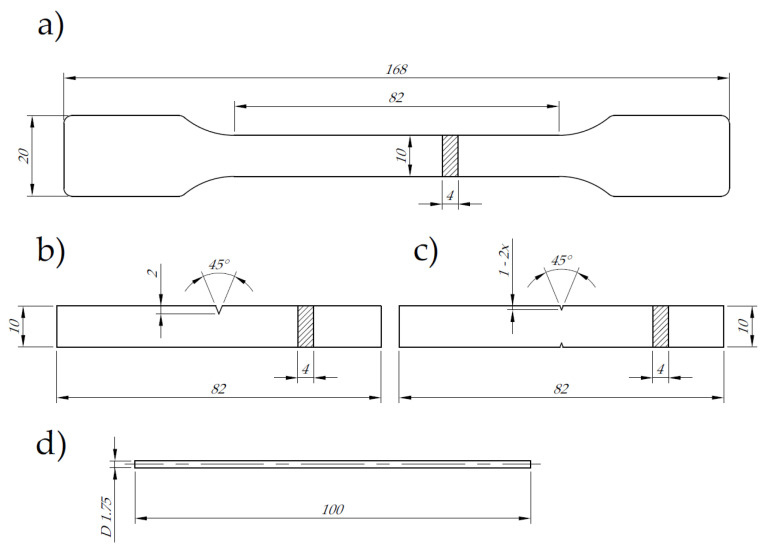
Test specimens used in the tests in accordance with the standards (dimension in mm): (**a**) static tensile strength (ISO 527), (**b**) Charpy impact strength (ISO 179), (**c**) impact tensile strength (ISO 8256), (**d**) filament sample.

As the test objects (modified printed structure), standard specimens compliant with the relevant standards were applied. Figure 1 shows the detailed dimensions of the test samples for individual tests. A filament (diameter 1.75 mm) with a length of 90 mm (measuring section 50 mm) was also tested.

### 2.2. Material

The material used in the tests was polylactide (PLA) filament by Spectrum Group Sp. z o. o. (Pęcice, Poland). The filament used in the research was characterized by a diameter of 1.75 ± 0.03 mm and melt flow index (MFI) of 6.10 ± 0.3 g/10 min (according to ISO 1133). The main factor determining the selection of PLA for research conduction was its predisposition for construction applications, its biodegradability and its ten-fold lower emissivity of harmful UFP particles (sizes below 100 nanometers) compared to ABS.

### 2.3. Research Stand

**Figure 2:**
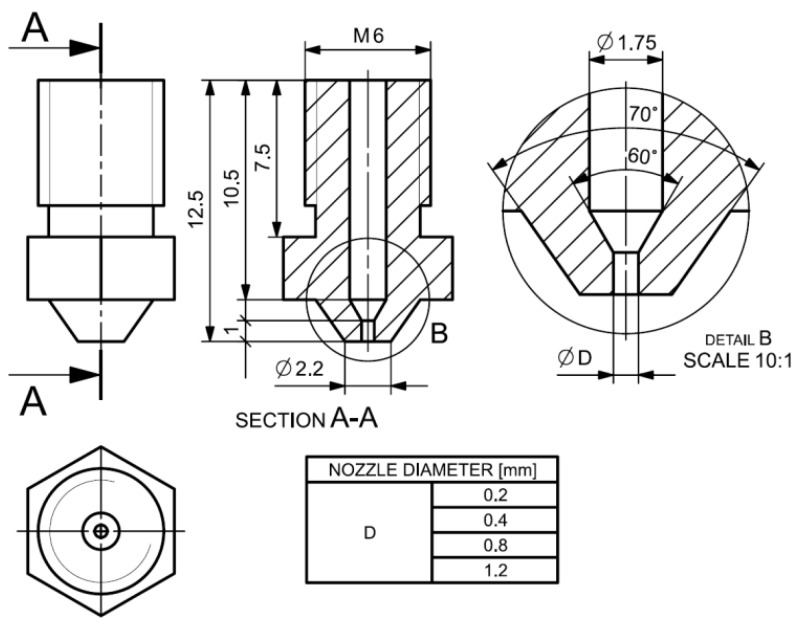
Geometry and basic dimensions of the print head nozzle.

A Tevo Tarantula 3D printer subjected to design modifications was used for the production of research samples. The modified structure allowed us to increase the stiffness of the entire kinematic system, which positively influenced the stability of the printing process. Moreover, the printer’s head was also modified to create the possibility of replacing the nozzle. This procedure allowed the changing of the nozzle’s basic geometrical feature, which is the diameter. Figure 2 shows the constant and variable dimensions of the nozzles used in the tests. The variable diameter of nozzle A took the following values: 0.2 mm, 0.4 mm, 0.8 mm and 1.2 mm.

### 2.4. Sample Preparation

The polylactide granulate used for the injection process was obtained by grinding the filament (cold cutting method) using a TS-10 granulator (IMPiB, Toruń, Poland). Injection molding was conducted with the use of an injection hybrid machine e-victory 310/110 (Engel GmbH, Schwertberg, Austria). The tool used for sample preparation was laboratory injection mold, which allowed us to produce standard test samples in accordance with PN-EN ISO 3167. Table 1 presents the process parameters applied in the injection molding process. The 3D-printed samples were prepared with the use of Repetier-Host software by Hot-World GmbH & Co. KG (Willich, Germany). The constant parameters of the 3D printing process can be seen in Table 2.

**Figure 3:**
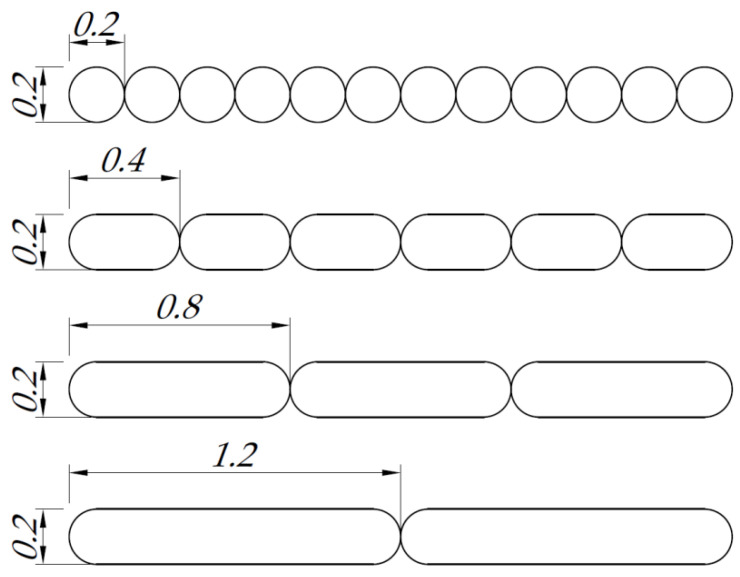
Influence of the diameter of the head nozzle on the overlapping of paths in individual layers.

The variable parameter in the research program was the diameter of the working nozzle, which assumed the following values: 0.2 mm, 0.4 mm, 0.8 mm and 1.2 mm. During the preparation of each sample, the cooling fan was turned on after the third printing layer was applied. The horizontal orientation of the samples in relation to the platform (the widest edge of the samples was placed along the X-axis of the printer’s build plate, following the load direction) was used. The use of a nozzle with a variable diameter allows the laying of paths with a greater degree of fusion and gives the possibility of obtaining a smaller number of horizontal connections between adjacent paths in individual layers (Figure 3). In this way, the structure characterized by the highest path width is closer to a solid structure than in the case of smaller nozzle diameters. Moreover, it was decided to maintain a constant layer thickness in order to obtain a constant contact area of adjacent paths.

**Figure 4:**
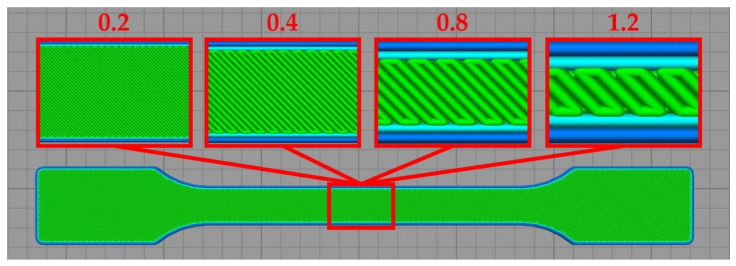
The arrangement of PLA paths in printed samples depending on the dimensions of the nozzle diameter used.

Figure 4 shows the arrangement of individual material paths in subsequent layers of the printed specimens. The samples produced by the FFF method were characterized by a path pattern of −45°/+45° to the direction of static and dynamic loads (the angle between paths of two successive layers was equal to 90°). For each nozzle diameter used, 100% of the filling degree was assumed. The dimensions of the V-type notches (beam-shaped samples) used in the impact tests are shown in Figure 1b,c. The geometry of the V-type notch was made with the use of a ZNO 2010 cutter (ZwickRoell GmbH & Co. KG, Ulm, Germany).

**Figure 5:**
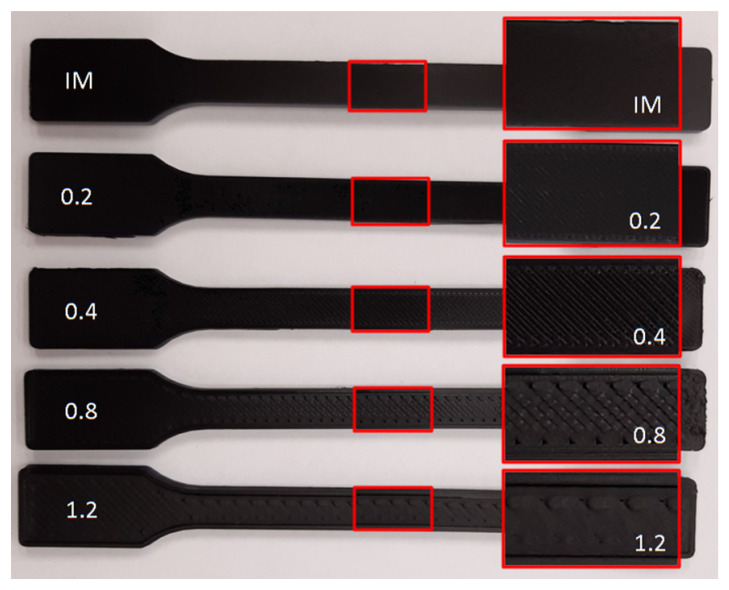
Filling structure of injection molding and 3D-printed samples.

Figure 5 presents the samples prepared for tensile tests, with the arrangement of paths in the measurement area shown. Images of the specimens produced with the use of different nozzle diameters are depicted. The same arrangement pattern was used in the case of bar samples.

### 2.5. Microscopic Analysis

The VHX-7000 microscope (equipped with a 3 MP camera) by Keyence (Osaka, Japan) was used to analyze the structures and fractures of the samples.

### 2.6. Density Analysis

The apparent density tests were carried out in accordance with the ISO 845 standard. The weight measurements were performed using a CPA225D-0CE (Sartorius Lab Instruments GmbH & Co. KG, Goettingen, Germany) laboratory scale with an accuracy equal to 0.01 mg. The volume was calculated based on the dimensions of the beam samples, which were measured with a Marcal 16 ER (Mahr GmbH, Goettingen, Germany) caliper with an accuracy equal to 0.01 mm.

### 2.7. Mechanical Tests

Tensile tests were carried out with the use of a Z030 (ZwickRoell GmbH & Co. KG, Ulm, Germany) testing machine. The test speed was 1 mm/min in the modulus determination phase and 50 mm/min until the end of the test. In order to determine the elastic modulus, a mechanical extensometer was used. The tensile strength of PLA filament with a diameter of 1.75 mm was determined under the same conditions as the injected samples in order to refer to the properties of the base material (PLA) and for comparative purposes.

**Figure 6:**
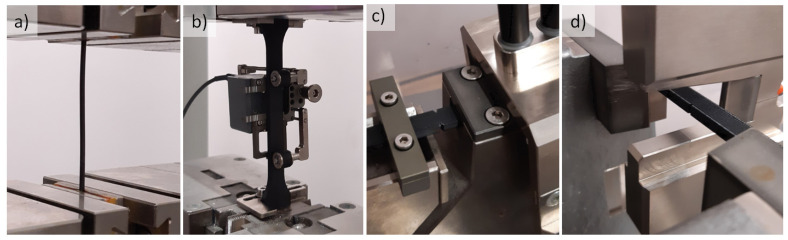
Research objects placed on testing stands: (**a**) filament, (**b**) dog-bone specimen, (**c**) impact tensile strength specimen, (**d**) Charpy impact test specimen.

The impact tests were carried out with the use of a HIT 50P (ZwickRoell GmbH & Co. KG, Ulm, Germany) impact tester. In the tensile impact tests, a hammer with an energy of 4 J was used. To determine the impact strength using the Charpy method, a hammer with an energy of 7.5 J was applied. Figure 6 presents the test objects mounted on sample holders of a static tensile tester and an impact tester.

## 3. Results and Analysis

### 3.1. PLA Mechanical Properties of the Injection-Molded Samples

The parameters of the material used were verified, and they are summarized in Table 3. Material parameters constituted a point of reference in the analyses presented in the paper. The test specimens were produced by the injection molding process (solid structure).

### 3.2. Structure Analysis 3D-Printed Objects

The relationships between the diameter of the nozzle and the measured geometrical features of the single paths are summarized in Table 4. In the case of the nozzle diameter of 0.2 mm, 0.147 ± 0.008 mm thick layers were obtained. Paths applied with the use of the 0.2 mm nozzle exceeded its diameter and had a 0.218 ± 0.009 mm width. In the case of the remaining values of the variable, the average layer thickness was close to the assumed value and ranged from 0.210 mm to 0.228 mm. In the case of 0.4 mm, 0.8 mm, and 1.2 mm nozzles, the path width was less than the size of the nozzle used and was equal to 86%, 90%, and 88% of its diameter, respectively.

**Figure 7:**
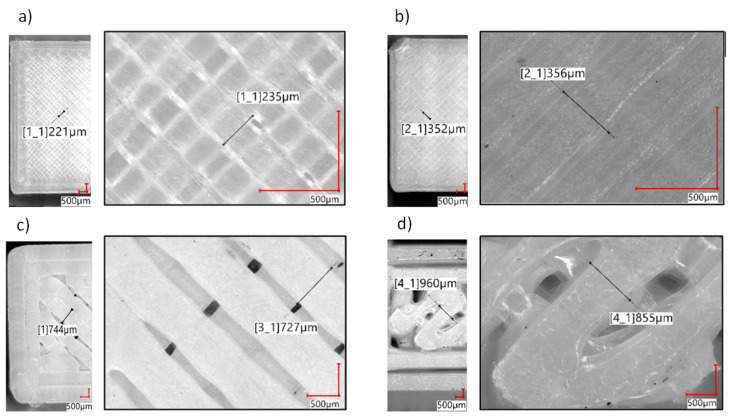
Microscope images of the outer layer of tested specimens with example path width measured: (**a**) 0.2 mm diameter nozzle, (**b**) 0.4 mm diameter nozzle, (**c**) 0.8 mm diameter nozzle, (**d**) 1.2 mm diameter nozzle.

An evaluation of the printed paths in the sample structure was performed on the outer layers of the samples. The outer layers were arranged in the same way as the rest of the underneath layers, which are not visible. The use of nozzles with the diameters of 0.2 mm and 0.4 mm results in solid tight layers being obtained (see Figure 7). In the case of larger nozzle diameters (0.8 mm and 1.2 mm), an openwork structure is obtained in the individual layers. Additionally, a change in the dimensions of the width of the path can be observed (Figure 6c,d).

**Figure 8:**
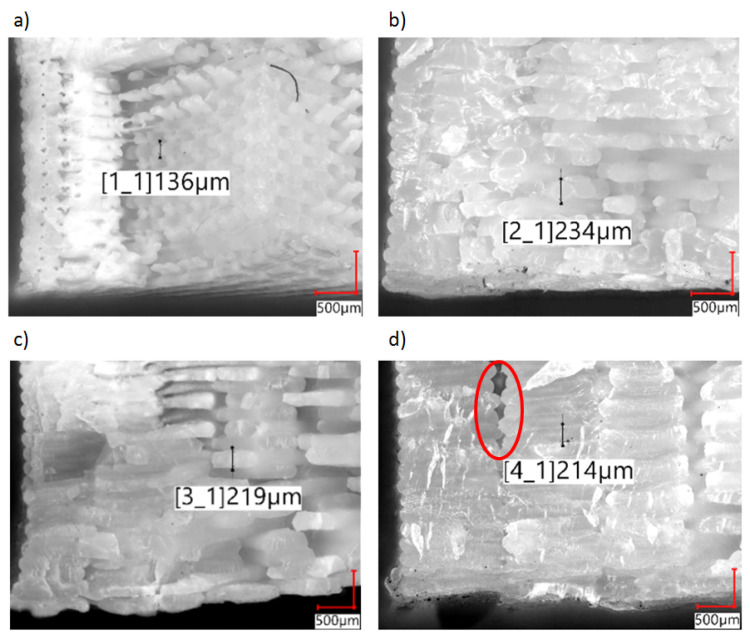
Microscope images of fracture of tested specimens with example layer thickness measured: (**a**) 0.2 mm diameter nozzle, (**b**) 0.4 mm diameter nozzle, (**c**) 0.8 mm diameter nozzle, (**d**) 1.2 mm diameter nozzle.

As a result of the use of smaller nozzles (diameters 0.2 mm and 0.4 mm), samples with an internal structure characterized by a greater order were obtained (fractures in Figure 8). In the case of the 0.2 mm nozzle, the sample structure shows regular voids (Figure 8a) between the layers and the outer and inner paths. The distinct separation of individual threads across the entire cross-section of the sample can be observed. In the structure of the test objects prepared with the use of 0.4 mm (Figure 8b) and 0.8 mm (Figure 8c) nozzles, areas of clear fusion, including the adjacent threads inside the layer and between the layers, can be observed. A distinct fusion between the threads inside a layer and extensive discontinuities in the structure (voids covering several layers) can be observed in the case of samples prepared with the use of the 1.2 mm nozzle (Figure 8d). As a result of a lack of connection between the outline and internal structure of the layer, repeated in different areas in subsequent layers (Figure 8d red circle marking), a notch is created. The resulting internal notch passing through several layers may have an impact on the deterioration of the results of the mechanical parameters obtained by both static and dynamic methods.

**Figure 9:**
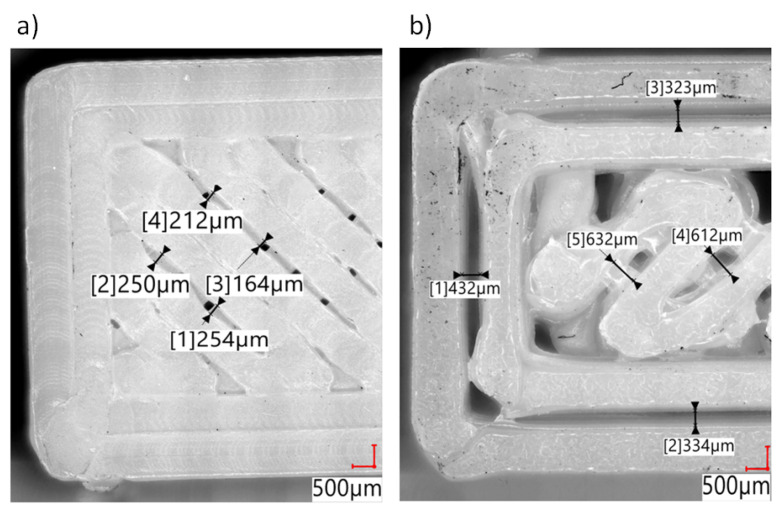
Microscope images of the outer layer of tested specimens with example path gap measured: (**a**) 0.8 mm diameter nozzle, (**b**) 1.2 mm diameter nozzle.

As a result of the use of 0.2 mm and 0.4 mm nozzles, a solid structure was formed (Figure 7a,b). This is a result of close contact between the adjacent paths within a single layer applied in the process. In the case of 0.8 mm and 1.2 mm nozzles, visible gaps between the threads were observed (Figure 9a,b). The size of the gaps ranged from 150 µm to 270 µm in the case of the 0.8 mm nozzle and from 300 µm to 800 µm for the 1.2 mm nozzle. In the case of using the nozzle with the diameter of 0.8 mm, the inner layers (without continuous contours) were connected pointwise. The test specimens printed with the 1.2 mm nozzle showed gaps between the solid outline and angular threads in the inner part of the layer.

**Figure 10:**
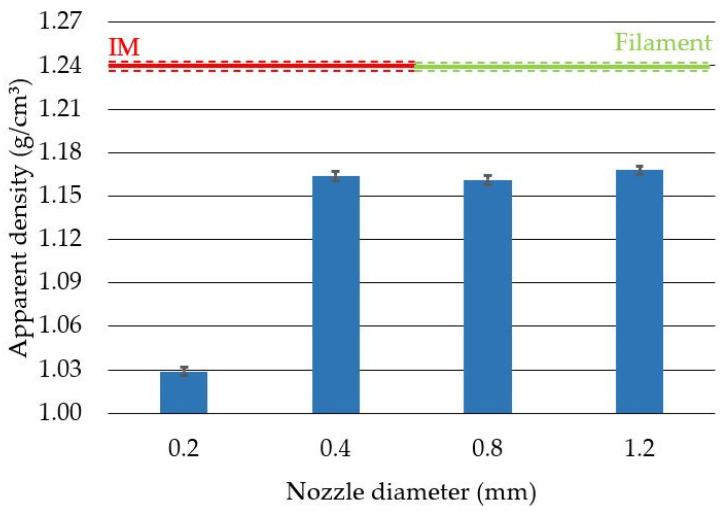
Average apparent density with standard deviation for samples produced with different nozzle diameters. IM—average value obtained for injected samples. Filament—average value for the filament before printing.

Apparent density measurements were performed to verify the amount of material used to produce the test specimens for each nozzle diameter. To determine the effect of the nozzle diameter on the apparent density of the produced samples, four variants were tested (Figure 10). As a reference point, the density of the injected samples and the filament were given. The lowest density was obtained for the nozzle with the diameter of 0.2 mm, which coincides with the study conducted by Triyono [28]. This result was caused by an incorrect ratio of the layer height to the nozzle diameter, which, in this case, was 1, while according to the adopted standards, this coefficient should not exceed 0.8 (recommended 0.5). Obtaining a factor equal to 1 results in larger technological voids in relation to the path width used, which can also be seen in Figure 6, Figure 7 and Figure 8. By maintaining the ratio of the layer height to the nozzle diameter below 0.8, a constant print density can be obtained, as shown by the results for 0.4 mm, 0.8 mm, and 1.2 mm nozzles.

### 3.3. Mechanical Characteristics

**Figure 11:**
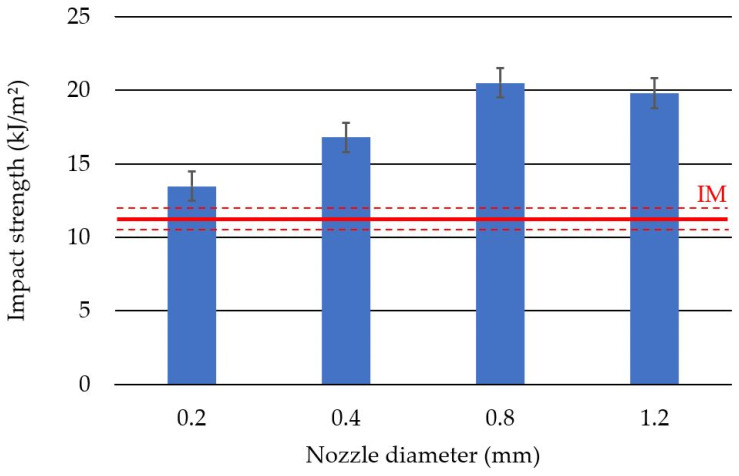
Average impact strength values with standard deviation for specimens produced with different nozzle diameters. IM—average value obtained for injected specimens.

Verification tests of objects manufactured using various production processes were carried out in the conditions of dynamic bending (Charpy’s impact test) and dynamic tensile (impact tensile test) tests. The highest impact strength value was obtained for the samples produced with the 0.8 mm nozzle and was equal to 20.55 ± 1.99 kJ/m^2^. The lowest value of impact strength was measured for the samples produced with the use of the 0.2 mm nozzle and was equal to 13.47 ± 1.8 kJ/m^2^. Despite the lowest result in comparison to the samples printed with the use of the remaining nozzle diameters, the observed impact strength was about 20% higher than in the case of the injection molding (Figure 11).

**Figure 12:**
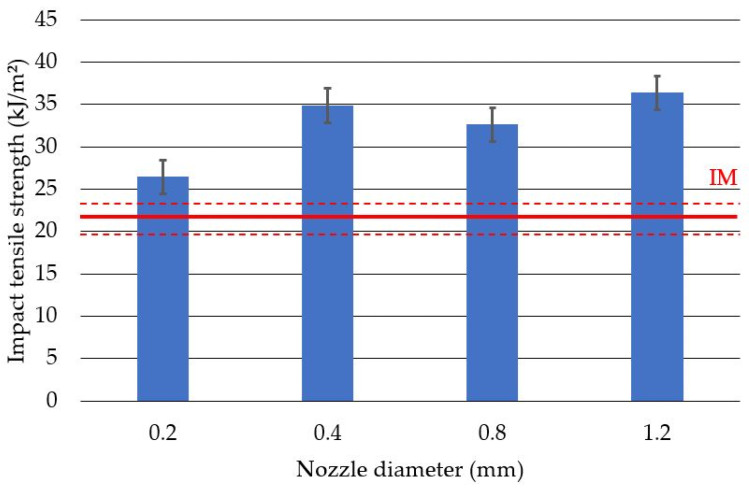
Average impact tensile strength values with standard deviation for specimens produced with different nozzle diameters. IM—average value obtained for injected specimens.

The highest value of the impact tensile strength was obtained for the samples produced with the 0.4 mm nozzle and was 36.43 ± 1.41 kJ/m^2^ (Figure 12). The lowest value of the tensile impact strength was measured for the samples produced with the 0.2 mm nozzle and was 26.46 ± 1.56 kJ/m^2^. In the case of both types of impact tests, the obtained results for the printed samples are higher than those obtained for the injection-molded ones. This conclusion is particularly well founded in the case of samples produced with the use of nozzles with diameters of 0.4 mm, 0.8 mm and 1.2 mm.

**Figure 13:**
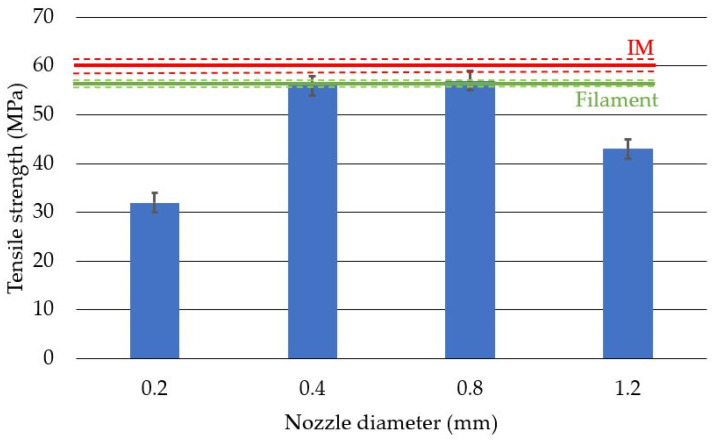
Average tensile strength values with standard deviation for samples produced with different nozzle diameters. IM—average value obtained for injected specimens. Filament—average value measured for single 1.75 mm filament fiber.

The results of the tensile tests are presented in Figure 13. Comparatively, the results for the samples produced by the injection method (red color) and the results for the filament (green color) are presented on the graph. In the case of 3D-printed samples with the use of nozzles with diameters of 0.4 mm and 0.8 mm, the tensile strength results were similar to those of the filament sample and were slightly lower than those produced by injection technology. The obtained results are similar to those of the Kuznetsov study [6]. The highest value of the tensile strength was determined for the samples produced with the use of the 0.8 mm nozzle and was equal to 56.6 ± 2.38 MPa. The lowest value of the tensile strength was measured for the samples produced with the 0.2 mm nozzle and was equal to 33.2 ± 2.19 MPa. The lowest value of Young’s modulus was measured for the samples produced with the use of the 0.2 mm nozzle and was 2.2 ± 0.11 GPa, while the highest value was achieved for the 0.4 mm nozzle and was equal to 3.15 ± 0.06 GPa. High tensile strength values (comparable to the results for the filament) obtained for 0.4 mm and 0.8 mm nozzle samples are caused by structures characterized by a high degree of fusion, where a small number of well-dispersed voids can be noticed. The lower values obtained for samples produced with the use of the nozzle with the diameter of 1.2 mm in relation to nozzles of 0.4 mm and 0.8 mm are due to the lack of connections between the paths (even several layers) of the contour and the internal lattice structure. The presence of an internal notch significantly weakens the cross-section under load (Figure 8d).

**Figure 14:**
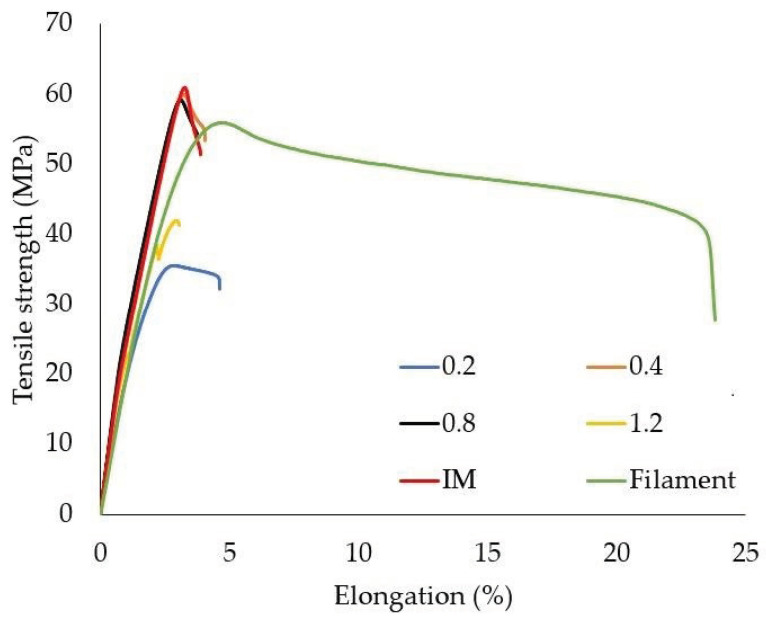
Selected example stress–strain curves recorded during static tensile tests.

Figure 14 presents compiled curves recorded during the static tensile test for the samples printed using various nozzle diameters, IM samples and filament samples. The curves obtained in the case of the 0.4 mm and 0.8 mm nozzles are the most similar to the curve registered for the injected sample. The different course of the tensile curve for the filament sample is due to the stacking of macroparticles, which allows for a higher elongation value. The elongation value is about 60% higher than the value of the other tested samples. In the case of the curve recorded for the 1.2 mm nozzle (yellow color), the moment of the break of the thick external paths arranged parallel to the direction of the load can be observed.

The specific strength (Nm/kg) of the samples was calculated, which was understood as the ratio of the mechanical tensile strength of the material (N/m^2^) and the apparent density (kg/m^3^). The highest specific strength was recorded for prints made with the 0.8 mm nozzle: 4.88 × 10^4^ Nm/kg. The value is slightly lower than the results of the injected sample which were equal to 4.89 × 10^4^ Nm/kg. The lowest value of the specific strength was found for samples produced with the 0.2 mm nozzle and was equal to 3.23 × 10^4^ Nm/kg. The specific modulus was calculated as the ratio of the modulus of elasticity (N/m^2^) and the apparent density (kg/m^3^). The values of the specific modulus for the printed samples were much higher (apart from the nozzle with a diameter of 0.2 mm) compared to the sample obtained with the injection technology.

**Figure 15:**
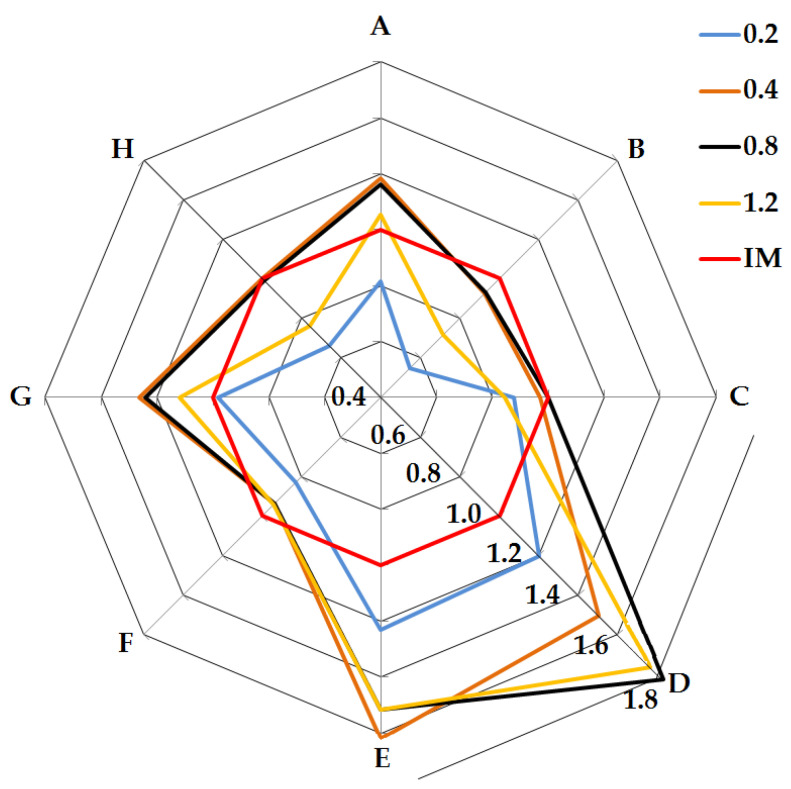
Comparison of mechanical properties of samples with a solid structure (injection molding) and 3D-printed samples. A—Young’s modulus; B—tensile strength; C—elongation; D—impact strength; E—impact tensile strength; F—apparent density; G—specific modulus; H—specific tensile strength.

In order to compare the results of the selected mechanical parameters obtained for the samples produced using the different technologies, a radar chart was used (Figure 15). The results of PLA samples produced by the injection method were used as the reference values of each property (values equal to 1.0 are marked with a red line). The reason for assuming such a reference point is the fact that the assessment of mechanical properties of polymeric materials is most frequently conducted with the use of injection-molded specimens. The favorable results of the impact tests (axes D and E) are due to the 3D-printed structure of the samples (polymer–gas structure). As a result of the structure’s response to the hammer’s energy inside the samples, the fibers between adjacent paths and layers are deformed, giving the effect of impact reinforcement. Positive effects are also obtained in the case of the parameters Young’s modulus (A axis) and the specific modulus (G axis) (similar influence of the printed structure) for samples printed with the use of nozzles with diameters of 0.4 mm, 0.8 mm and 1.2 mm. For nozzles with diameters of 0.4 mm and 0.8 mm, the reduction in the apparent density positively influenced the specific strength values, which are comparable to the values of the injected samples (H axis). The poor tensile strength results, and, thus, the specific strength results for samples produced with the 1.2 mm nozzle, are due to significant discontinuities in the structure between the layers (Figure 8).

## 4. Discussion

The width of the overlaid path and process voids are observed in many publications on 3D printing. However, an attempt to measure these values and contrast them with the rest of the printing process parameters and mechanical properties is rarely made. 

The average apparent density of the additively manufactured samples obtained for the 0.2 mm nozzle was 17% lower than the value obtained for injected samples and the filament. However, in the case of the 1.2 mm nozzle, this value was 6% lower than the average apparent density of the injected samples and the filament. The difference in the average density between the 0.2 mm and 1.2 mm nozzles was 12%. The trend of increasing sample density with increasing nozzle diameter was confirmed in Triyono’s study [28]. In their study, the authors showed that a doubling of the nozzle diameter results in a 4% increase in density.

The highest average Charpy impact strength was achieved for the samples manufactured with the 0.8 mm nozzle and was equal to 20.55 kJ/m^2^. The results obtained were similar to those of the study conducted by Schiavone [29]. In Schiavone’s work, great emphasis was placed on the thermal aspects of the processed material. The work conducted showed that better results can be obtained by arranging the samples differently in the printer workspace.

The static tensile tests showed that the results obtained for the specimens printed with 0.4 mm and 0.8 mm nozzles achieved values close to the reference value, i.e., injection-molded specimens and the manufacturer’s declared value. In the work of Yao [30], PLA samples were printed with the use of various angles in relation to the working platform in similar conditions to ours. The tensile strength obtained by the authors for a 0.2 mm layer height was equal to 53.08 MPa, which is 12% lower than the value obtained in our study. In the work of Hanon [31], various alignments of test samples were used, similarly to their predecessors. In the comparison of the test results, the arrangement of the samples corresponding to the setup used in our study was considered. The tensile strength investigated by Hanon reached a value of 55 MPa, which was 7% lower than the value obtained in the conducted tests. In the work of Kuznetsov [6], it was shown that a constant layer height and an increase in the nozzle diameter have a positive effect on the mechanical strength of printed parts—they increase it. The authors proved that a low parameter of layer height to nozzle diameter causes a significant improvement in mechanical strength and significantly affects the size of the technological voids.

## 5. Conclusions

The research showed an influence of the nozzle diameter on the macrostructure and selected strength properties of the analyzed samples. A microscopic analysis of the fractures of the samples showed that in the case of nozzle diameters greater than 0.4 mm, an increase in the share of air voids between the print paths occurred. This phenomenon may have an impact on the impact strength value of the test specimens, as the highest impact strength values were recorded for samples with the largest voids between the paths of the polymer material.

The voids in the sample structure may absorb some of the energy generated during the impact test. Moreover, in the case of the strength properties, the samples produced with the use of nozzles with diameters of 0.4 mm and 0.8 mm were characterized by a higher tensile strength. 

The lowest values of mechanical properties were observed in the case of samples produced with the use of the 0.2 mm nozzle. In this case, the diameter of the nozzle was equal to the assumed height of the print layer. The appropriate layer height should be selected based on the nozzle diameter. The ratio of the layer height to the nozzle diameter should not exceed 0.8. In the case of using larger diameters of the extruder nozzle, maintaining a constant layer height at the level of 0.2 mm resulted in better conditions for melting each subsequent layer during the contact of the hot extrudate with the previous print layer. It is likely that this phenomenon has a decisive influence on the formation of interlayer adhesion of the produced samples.

The correct selection of the head type enables one to obtain satisfactory functional properties of products, both in terms of strength and surface effects, similar to the values characterizing the elements manufactured in mass technologies. It is expected that further studies will be preceded by a detailed analysis of the rheological properties of the polymers used and their relationship with the adhesion phenomena occurring between subsequent paths of the printed filament. In future research, the nozzles of the head with the maximum possible diameter for a filament with a diameter of 1.75 mm will be used (obtaining the maximum path width). The algorithm deciding the density of the paths will also be corrected in order to obtain the most solid structure. Modifying the algorithm (compacting the arrangement of paths) in order to adjust the printed structure for the use of a nozzle with a diameter of 1.2 mm would allow one to eliminate the effect of structure discontinuity (no occurrence of notches) and to obtain much more favorable results with static/dynamic methods.

## Figures and Tables

**Table 1:** The constant parameters of the injection molding process.

Parameter	Value
plasticizing zones temperature (°C)	230/230/200/160/40
injection rate (cm^3^/s)	100
mold temperature (°C)	30
shot volume (cm^3^)	76
packing pressure (MPa)	50
packing time (s)	34
cooling time (s)	30
counter pressure (MPa)	5

**Table 2:** The constant parameters of the printing process.

Printing Parameter	Value
nozzle temperature (°C)	230
platform temperature (°C)	70
printing speed (mm/s)	20
layer thickness (mm)	0.2
path angle (°)	−45/+45
infill (%)	100
fan speed (%)	50

**Table 3:** The properties of used PLA material.

Material Parameter	Value
Young’s Modulus (MPa)	2702 ± 38
Tensile strength (MPa)	60.0 ± 1.1
Elongation at break (%)	3.20 ± 0.03
Impact strength (kJ/m^2^)	11.24 ± 1.86
Impact tensile strength (kJ/m^2^)	21.5 ± 1.8
Apparent density (g/cm^3^)	1.24 ± 0.03
Specific modulus ((N·m)/kg)	2.18 × 10^6^ ± 0.03 × 10^6^
Specific tensile strength ((N·m)/kg)	4.88 × 10^4^ ± 0.07 × 10^4^

**Table 4:** Optically measured geometrical features of manufactured samples in the function of nozzle’s diameter.

Nozzle Diameter (mm)	Average Layer Thickness (mm)	Average Path Width (mm)
0.2	0.147 ± 0.008	0.218 ± 0.009
0.4	0.228 ± 0.009	0.347 ± 0.003
0.8	0.218 ± 0.019	0.720 ± 0.031
1.2	0.210 ± 0.007	1.063 ± 0.019

## Data Availability

Not applicable.
